# Anthropogenic climate change is worsening North American pollen seasons

**DOI:** 10.1073/pnas.2013284118

**Published:** 2021-02-08

**Authors:** William R. L. Anderegg, John T. Abatzoglou, Leander D. L. Anderegg, Leonard Bielory, Patrick L. Kinney, Lewis Ziska

**Affiliations:** ^a^School of Biological Sciences, University of Utah, Salt Lake City, UT 84112;; ^b^Management of Complex Systems Department, University of California, Merced, CA 95343;; ^c^Department of Integrative Biology, University of California, Berkeley, CA 94720;; ^d^Department of Evology, Evolution, and Marine Biology, University of California, Santa Barbara, CA 93106;; ^e^Center for Environmental Prediction, Rutgers University, New Brunswick, NJ 08854;; ^f^Department of Medicine, Allergy and Immunology and Ophthalmology, Hackensack Meridian School of Medicine, Nutley, NJ 07110;; ^g^New Jersey Center for Science, Technology and Mathematics, Kean University, Union, NJ 07083;; ^h^School of Public Health, Boston University, Boston, MA 02118;; ^i^Mailman School of Public Health, Columbia University, New York, NY 10032

**Keywords:** climate change, respiratory health, detection, attribution, Earth system model

## Abstract

Airborne pollen has major respiratory health impacts and anthropogenic climate change may increase pollen concentrations and extend pollen seasons. While greenhouse and field studies indicate that pollen concentrations are correlated with temperature, a formal detection and attribution of the role of anthropogenic climate change in continental pollen seasons is urgently needed. Here, we use long-term pollen data from 60 North American stations from 1990 to 2018, spanning 821 site-years of data, and Earth system model simulations to quantify the role of human-caused climate change in continental patterns in pollen concentrations. We find widespread advances and lengthening of pollen seasons (+20 d) and increases in pollen concentrations (+21%) across North America, which are strongly coupled to observed warming. Human forcing of the climate system contributed ∼50% (interquartile range: 19–84%) of the trend in pollen seasons and ∼8% (4–14%) of the trend in pollen concentrations. Our results reveal that anthropogenic climate change has already exacerbated pollen seasons in the past three decades with attendant deleterious effects on respiratory health.

## Significance

Human-caused climate change could impact respiratory health, including asthma and allergies, through temperature-driven increases in airborne pollen, but the long-term continental pollen trends and role of climate change in pollen patterns are not well-understood. We measure pollen trends across North America from 1990 to 2018 and find increases in pollen concentrations and longer pollen seasons. We use an ensemble of climate models to test the role of climate change and find that it is the dominant driver of changes in pollen season length and a significant contributor to increasing pollen concentrations. Our results indicate that human-caused climate change has already worsened North American pollen seasons, and climate-driven pollen trends are likely to further exacerbate respiratory health impacts in coming decades.

Human-caused climate change is expected to have widespread negative impacts on public health through a range of pathways (1–3). Climate change could trigger spatial and temporal shifts in plant airborne pollen loads, which have major respiratory health consequences for allergies and asthma (4–7), viral infections (8), school performance and downstream economic impacts (9), and emergency room visits (5, 10). Because pollen concentrations are often highly temperature-sensitive (11, 12), anthropogenic climate change could substantially harm respiratory health by increasing pollen concentrations and/or lengthening pollen seasons and exposure times (13–15). Thus, understanding the spatial and temporal variation in pollen loads and whether anthropogenic climate change is a major contributor to such changes at large geographical (e.g., continental) scales is urgently needed to estimate potential changes in respiratory health.

Climate change detection and attribution analysis is a powerful tool for linking long-term climate change and observed impacts (16, 17). However, detection and attribution techniques have not been widely applied to public health impacts, despite major implications for policy and public health interventions (18). Detection and attribution approaches provide a substantial advance by connecting societal impacts to ongoing climate change and rigorously quantifying the role of human forcing of the climate in trends of impacts (18). Detection and attribution approaches aim to statistically detect whether a variable/impact is changing and attribute how much of the observed change was contributed by anthropogenic climate change (19–21).

Among climate-related health impacts, pollen trends may be particularly suited to detection and attribution because both elevated temperature and CO_2_ concentrations have been found to increase pollen production in greenhouse or growth chamber experimental studies (22–25). A few long-term observational studies on selected plant taxa or at a small number of sites have found increases in pollen concentrations and pollen season length over time, often correlated with temperature (11, 12, 15, 26), although temperature–pollen season relationships may depend on chilling requirements in some taxa (27, 28). Yet a continental-scale detection of long-term pollen trends with a formal attribution to anthropogenic climate change is lacking.

Here, we leverage a continental-scale dataset of long-term pollen records from 60 North American cities spanning 1990–2018 (821 site-years of data; *SI Appendix*, Table S1), observational climate datasets, and a suite of simulations from 22 Earth system models to conduct a detection and attribution analysis on spatial and temporal characteristics of aero-allergenic pollen trends. We ask: 1) What are the long-term trends in common pollen metrics; i.e., can trends be detected in various estimates of pollen season severity? 2) Do climate—temperature and precipitation variables—and/or increasing atmospheric CO_2_ concentrations play a prominent role in driving interannual variation and trends in pollen metrics? 3) How much of the observed temporal trends in pollen metrics can be attributed to recent human-caused changes in climate?

## Results and Discussion

**Fig. 1.**
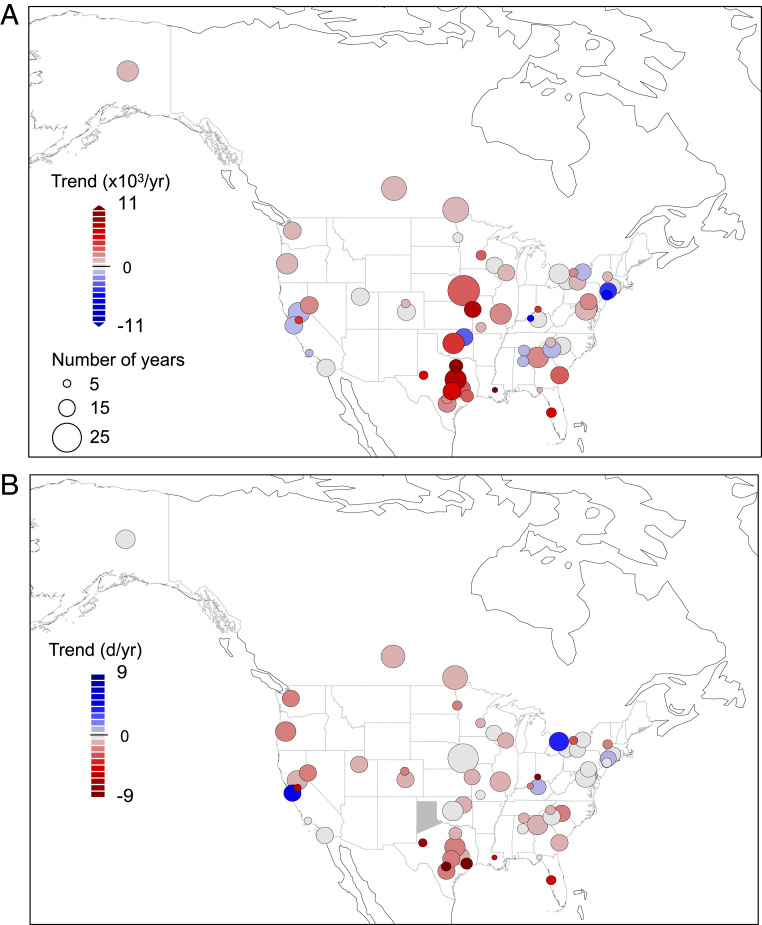
Detection of long-term worsening of pollen seasons in North America. Linear trend over individual stations of the annual pollen integrals (*A*) and pollen season start date (*B*) across the 60 pollen stations in North America. Warm colors indicate increasing annual pollen integrals or earlier start dates and circle size is proportional to the years of data at each station.

We detected significant temporal trends in 7 of 10 common pollen metrics, including daily pollen extremes, pollen season start date and length, and seasonal and annual total pollen integrals, that revealed a substantial intensification of pollen seasons in North America over the 1990–2018 period (*SI Appendix*, Tables S2 and S3). We observed widespread temporal increases of 20.9% and 21.5% in annual and spring (February–May) pollen integrals, respectively, between 1990 and 2018 (*P* < 0.0001 for both metrics; Fig. 1 and *SI Appendix*, Table S3). The largest and most consistent increases were observed in Texas and the midwestern United States (Fig. 1*A*). Among taxa, tree pollen showed the largest increases in spring and annual integrals (*SI Appendix*, Fig. S1). These continental trends were robust to sensitivity analyses around the number of stations included (*SI Appendix*, Table S2) and the longevity of station observations (*SI Appendix*, Fig. S2). We also found significant advances of ∼20 d in pollen season start date and lengthening of the pollen season by ∼8 d over the same period (*p*_start_ = 0.01; *p*_length_ = 0.0003; Fig. 1*B*). Advances in pollen season start date and increases in spring pollen integrals strongly support a phenological seasonal shift of pollen loads to earlier in the year (*SI Appendix*, Table S3 and Fig. S3). Long-term increases in pollen season length and annual pollen integrals indicate that exposure times to allergenic pollen as well as amount of pollen have increased significantly for North America in recent decades.

**Fig. 2.**
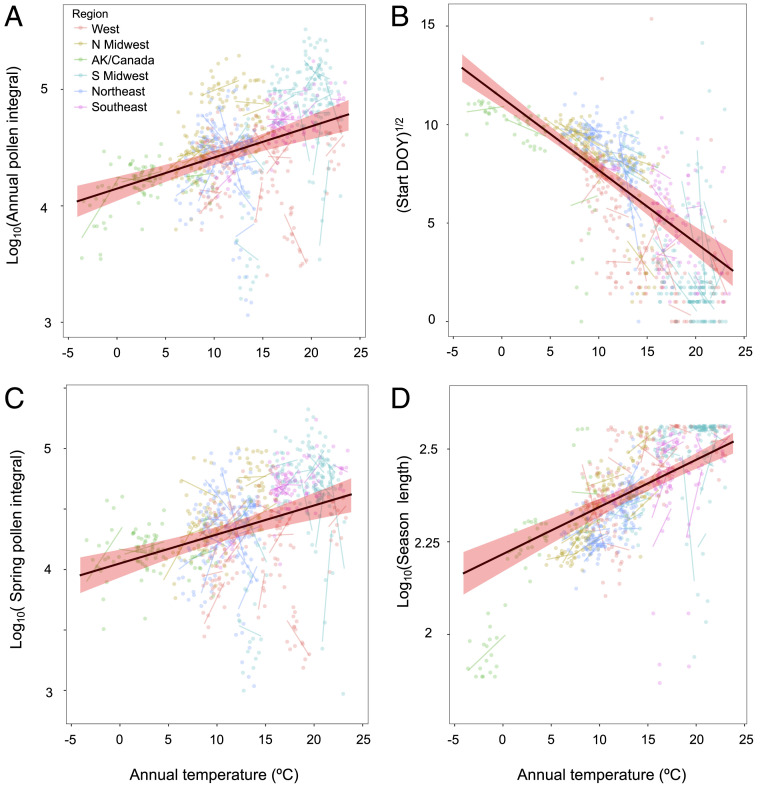
Temperature strongly influences pollen seasons and loads in North America. Predicted slope from linear mixed effects models across 60 North American cities of annual average temperature against the total annual pollen integrals (*A*), pollen season start date (DOY) (*B*), total spring (February–May) pollen integrals (*C*), and total pollen season length (days) (*D*). Points are individual years at individual stations. Thin lines are station-level trends. Point/line colors are regions (*SI Appendix*, Fig. S5). Shaded areas indicate the 95% CI of the fixed effect.

We conducted a model selection analysis to quantify the climate drivers of the four most important pollen metrics: annual pollen integral, spring pollen integral, pollen season start date, and pollen season length. We tested eight annual and seasonal climate variables, including temperature, precipitation, frost days, and atmospheric CO_2_ concentrations in a mixed-effects model framework to account for city-to-city variation. We found that mean annual temperature was the strongest predictor of these four pollen metrics (*P* < 0.0001 for all metrics; Fig. 2 and *SI Appendix*, Table S4). The full mixed-effects models explained 51–90% of the variance (i.e., conditional *R*^2^) in pollen metrics, and mean annual temperature alone (i.e., marginal *R*^2^) explained 14–37% of the variance in pollen metrics (*SI Appendix*, Figs. S4 and S5 and Table S4). Notably, while atmospheric CO_2_ concentrations were sometimes included in the group of the most parsimonious models, the variation explained was often quite low (e.g., annual integral *R*^2^_marginal_ = 0.01). This indicates that while an impact of CO_2_ concentrations can be detected, consistent with experimental greenhouse studies, temperature appears to be a much stronger driver of pollen variability in space and time at continental scales.

We used climate model output from 22 Earth system models (14 models from the Coupled Model Intercomparison Project [CMIP] Phase 5 and 8 models from CMIP Phase 6; *SI Appendix*, Table S5) to calculate the signal of anthropogenic forcing of the climate system on pollen integrals modeled as a function of mean annual temperature. This approach enables attribution of how much human forcing of the climate system has influenced trends in pollen variables. We calculated the anthropogenic contribution in the full 1990–2018 record and the more recent 2003–2018 period where data were available from at least 40 pollen stations across North America.

**Fig. 3.**
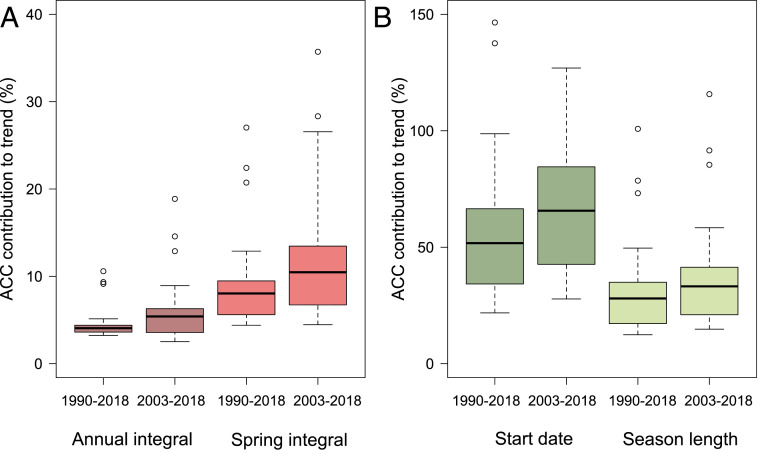
Anthropogenic climate change (ACC) has exacerbated pollen seasons. Boxplot of the percentage contribution of ACC to the long-term (1990–2018) and more recent (2003–2018) trends of annual total pollen integrals (dark red) and spring total pollen integrals (red) (*A*) and pollen season start date (dark green) and season length (light green) (*B*) across 60 pollen stations in North America. Data are plotted from 22 climate models (i.e., each model’s estimated fractional contribution to the observed continental trend from the mixed effect model).

Anthropogenic climate change was a strong driver of trends in pollen season metrics and a more modest driver of trends in pollen integrals. Anthropogenic forcing contributed to an estimated 35–66% (interquartile range) of the full trend and 45–84% of the recent trend in pollen season start date and 19–35% and 22–41% of the trend in pollen season length over the 1990–2018 and 2003–2018 periods, respectively (Fig. 3). Human forcing contributed 4–8% (mean_2003–18_ 6.5%) of the trend in annual pollen integral and 4–14% (mean_2003–18_ 12%) of the long-term trend in spring integral (Fig. 3). The anthropogenic contribution was stronger in the more recent 2003–2018 period than in the full 1990–2018 period (Fig. 3), likely due both to an increasing signal from anthropogenic forcing over time and to increased ability to detect the signal in the more recent period due to more stations. The anthropogenic signal appears to be more modest in annual integrals compared to spring integrals due to a “seasonal compensation” effect whereby some decreases in summer pollen integrals may dampen the total annual signal (*SI Appendix*, Table S3), likely indicative of shifting phenology of plant species to earlier in the year in many regions (14, 29, 30).

Our results demonstrate that human forcing of the climate system has substantially exacerbated North American pollen seasons, particularly for pollen season duration and spring pollen integrals. These findings can also inform ongoing efforts to include prognostic pollen models within Earth system models to make spatial and temporal projections of pollen seasons under future climate scenarios (31) and when combined with seasonal and near-term climate forecasts may enable seasonal pollen forecasts, similar to crop yield forecasts (32). We note that these anthropogenic contributions are likely conservative estimates, given that the influences of climate on pollen are complex, and this analysis considers all taxa’s pollen combined and temperature-driven interannual variability. Other potential climate and nonclimate drivers could potentially play a role as well, including changes in urban vegetation patterns, species composition, and biomass.

Allergies and asthma are responsible for substantial morbidity burdens and associated medical costs in the United States (33). Long-term data show significant increases in allergen sensitivities (both prevalence and number of allergens) across all age groups in the United States, with trends of increasing pollen sensitization in childhood leading to increased adolescents and adults with allergic asthma (34). Pollen is an important trigger for many allergy and asthma sufferers, and pollen concentrations are strongly linked to both medication purchases and emergency hospital visits (5, 6), as well as susceptibility to viral infections through exacerbating respiratory inflammation and weakening immune responses (8). Thus, while not analyzed directly here, we hypothesize that climate-driven changes in spring and/or annual pollen integrals would have important implications for spatial and temporal patterns of allergy and asthma prevalence and associated medical costs.

These data represent a continental-scale detection and attribution of anthropogenic climate change on long-term pollen trends. Detection and attribution of climate impacts is a rapidly growing field (19), and detection and attribution of public health-related climate impacts is an urgent research area (1, 18). While the pollen-respiratory health linkages are complex, our results highlight that longer pollen seasons and higher pollen concentrations are being driven in part by human-driven temperature increases and are increasing the risks of respiratory health problems in real time. Thus, a clearly detectable and attributable fingerprint of human-caused climate on North American pollen loads provides a powerful example of how climate change is contributing to deleterious health impacts through worsening pollen seasons.

## Methods

### Pollen Data and Temporal Trend Detection.

We compiled pollen concentrations collected by the American Academy of Allergy, Asthma, & Immunology (AAAAI)–National Allergy Bureau’s (NAB) pollen count stations. Airborne pollen is sampled over a 24-h period at NAB stations typically using a Rotorod Sampler or Burkard spore trap and then counted by trained experts (35). Although the aerial sampling methods differ in their collection efficiency of different pollen sizes, the statistical methods we use throughout the study (mixed effects models) include station-level random slopes and intercepts to enable robust estimates of temporal trends and climate relationships across all stations. NAB stations disaggregate pollen concentrations to varying degrees by taxa. We primarily analyzed all taxa combined (total pollen) to be able to include the largest number of NAB stations in the analysis but performed one sensitivity analysis examining broad categories of tree, grass, and weed taxa for important seasonal and annual integrals (*SI Appendix*, Fig. S1). NAB stations are considered to provide accurate and rigorous estimates of pollen concentrations and have been used in previous observational studies (12, 26).

We set several a priori criteria for pollen stations and years to be included in the analysis. Because we aim to assess long-term trends, we only included stations with five or more years of data. For each station, each year had to contain at least 10 measurements, although most station-year combinations contained extensive measurements within a year (mean and median measurements/year: 168 and 163, translating to a measurement on average every 2–3 d; *SI Appendix*, Fig. S6 and Table S1). No trend in the number of measurements per station over time was detected (*P* = 0.77; *SI Appendix*, Fig. S6). A total of 57 NAB stations met our criteria and we additionally added 3 stations that met our criteria from a recently published observational study (12) that included data in Canada and Alaska, bringing our total station count to 60. Next, we checked the records for each station for internal consistency by plotting individual stations over time. We examined these plots to check if any stations had inconsistent jumps within or between years or potential “missing value” issues where the main pollen season might have been missed in a given year (e.g., daily concentration measurements start in April when previous years show pollen spikes in March). We observed four station-year combinations spread across two stations (stations 58 and 59; *SI Appendix*, Table S1) with potential inconsistencies and removed those station-years.

Then, for each station-year combination, we linearly interpolated 24-h concentration measurements over the course of the year. This method yielded daily concentrations for each station and year that most parsimoniously ingests all available 24-h concentration data and accommodates stations with different measurement frequencies (e.g., every 2 d vs. every 5 d). We compared this method to a similar approach in a previous study that used weekly aggregation (12) at three common cities and observed strong agreement in the annual integral estimates (*R*^2^ = 0.94). We then calculated 10 pollen metrics from the daily time series. We calculated the maximum, mean, and median of all daily concentration values in a year for a station. To look at pollen season lengths, we set a threshold based on diagnostic plots that the pollen season starts on the first day of the calendar year when daily concentrations exceeded the 30th percentile of raw daily concentration measurements for that station and ends when the last daily concentration falls back below that threshold. We did a sensitivity analysis and observed very similar patterns when varying the threshold from 20th to 40th percentile. From this threshold, we estimated the “start date,” “end date,” and “season length” of the pollen season each year. Finally, we calculated seasonal and annual pollen integrals by summing the daily concentrations of February 1–May 31 for spring integrals, June 1–August 31 for summer integrals, September 1–November 30 for fall integrals, and January 1–December 31 for annual integrals. Daily data were not available to us for two of the Canadian stations from the previous study (12) and for those two stations we estimated spring pollen integrals using the strong relationship between spring and annual integrals as in the Fairbanks, AK, station (*R*^2^ = 0.96).

We note as a caveat that NAB pollen measurements often employ a convenience sampling strategy where measurements are collected for only part of the year in many locations, based on a historical understanding of the pollen season at that location, and often only on certain days of the week. This sampling approach increases the uncertainty in daily pollen concentration metrics (e.g., mean or maximum concentrations) and pollen season start and end dates. We conducted two sensitivity analyses to test the potential for this seasonal sampling strategy to influence our primary results. First, we flagged station-year combinations where the first pollen measurement was above the 30th percentile, indicating the station might have missed the true start of the pollen season. All of our central metrics’ temporal trends were robust to excluding those data (*P* < 0.01 for all). Second, we tested for systematic biases over time in the distribution of the initial pollen concentration measurement or first five measurements of each station-year combination, which would indicate a widespread pattern of missing the start of the pollen season if the earlier part of each station’s record tended to miss the start of the season more frequently. We found no significant differences between the first and second halves of stations’ records (*P* > 0.15, *SI Appendix*, Fig. S7). These indicate that the seasonal sampling strategy is unlikely to greatly bias our results on pollen season start dates.

To quantify temporal changes in pollen metrics across all stations (i.e., detection in the detection and attribution framework), we used linear mixed effects models of pollen metrics versus year. Mixed effects models are particularly useful and appropriate for this analysis because they allow an analysis of the full dataset (avoiding challenges of multiple hypothesis testing from individual regressions/tests for each station) while incorporating a random effect for station that allows for station-specific slope, intercept, and variance terms. This random effect of station accounts for unobserved station-to-station variation, for instance in sampling method or expert counter differences, while rigorously estimating a global effect across all stations. We used the same model structure determined in the climate-pollen analysis (see below) of a random slope, intercept, and variance across stations and log-transformed pollen variables (see *Statistics*). As sensitivity analyses, we conducted this temporal trend analysis for annual integrals including all station-years versus only those station-years with >5 or >40 stations collecting data. We also tested all station-years versus data only from stations with 10+ years of data. We observed similar results with all data subsets (*SI Appendix*, Table S2 and Fig. S2). For spatial visualization in Fig. 1, we plot the linear slopes from ordinary least squares linear regression, but the rigorous trend detection used in the detection and attribution analysis was based on the mixed effect model estimation of temporal slope (fixed effect) across all stations.

We examined regional differences in pollen trends but did not observe substantial differences across regions in the main pollen metrics (*SI Appendix*, Fig. S8). Because statistical power fell rapidly at a regional level due to limited stations within certain regions, we did not conduct further regional-level analyses.

### Climate Data.

We used gridded climate data from the University of East Anglia’s Climatic Research Unit. We extracted monthly temperature, precipitation, and frost days from the HadCRUT4 dataset at 0.5° spatial resolution (36) and calculated monthly time-series of these variables from 1991 to 2018 over all 60 pollen stations. We used annual atmospheric CO_2_ concentration data from the National Oceanic and Atmospheric Administration’s Mauna Loa Observatory over that period (37). For each station and year, we calculated mean annual temperature, total annual precipitation, mean spring (February–May) temperature, total spring precipitation, total number of spring frost days as a coarse estimate of chilling requirements, mean summer temperature, total summer precipitation, and total number of summer frost days. This yielded eight climate metrics in total, plus atmospheric CO_2_ concentrations, to be used as predictor variables in the pollen-climate models below.

### Pollen-Climate Models.

To estimate the effect of climate on pollen concentrations and integrals, we conducted a model selection analysis using mixed effects models on the seven pollen metrics with significant temporal trends detected (*SI Appendix*, Table S3). Following recommended practice on mixed effects model selection (38), we first included all nine predictor variables as fixed effects and determined the model structure of random effects and if transformations were needed. Log_10_ transformations were needed on all variables, except for pollen season start date where a square-root transform was best. Model structure of random effects was determined both by Akaike Information Criterion (AIC) scores and quantile-quantile plots. The most parsimonious model by AIC and the model that yielded robust QQ plots was a random slope and intercept, allowing for unequal variances across stations.

We then used an “all possible models” model selection technique whereby all potential combinations of predictor variables (nine predictors) are used as fixed effects in the model and ranked using AIC scores to generate the most parsimonious model or group of models. This approach tends to be more robust than forward or backward model selection. The intercept-only model (i.e., no climate predictors) was the best model for three pollen metrics (maximum daily concentration, mean daily concentration, summer total integral) and we explored those metrics no further. This led to the four prominent pollen metrics—spring integral, annual integral, pollen season start date, and pollen season length—upon which we focused all subsequent analyses. Using the table of AIC-ranked models, we further examined the marginal and conditional *R*^2^ values of all models within AIC values within 3 from the top model (*SI Appendix*, Table S4). AIC values of 3 or more are generally considered to be strong evidence for favoring one model over another and, thus, models within 3 AIC are all potentially plausible (39). Within these top models, we chose the model with the highest marginal *R*^2^ value, indicating that the fixed effects can explain the highest amount of variance in the model. For all four pollen metrics, this model included only mean annual temperature (*SI Appendix*, Table S4).

### Earth System Models and Detection-Attribution Analysis.

We downloaded monthly surface air temperature (variable “tas”) climate model output from 14 Earth system models in the CMIP5 and 8 Earth system models in the CMIP6 databases (40, 41) (*SI Appendix*, Table S5). For each model, we downloaded the Historical (all forcings) simulation and representative concentration pathway (RCP) 8.5 (SSP5–8.5 in CMIP6) for the first ensemble member. One model in CMIP6 (BCC-CSM2) did not have SSP5–8.5 simulations uploaded when we downloaded data and, thus, we used the similar SSP3–7.0 scenario simulation for that model. While there is much uncertainty regarding projected pathways, the pathways chosen have little bearing on the contemporary signal of anthropogenic forcing that we focus on. We then regridded all climate models to a common 1° × 1° resolution, calculated the mean annual temperature for each year, and following common detection and attribution practices, subtracted the 1850–1880 climatology mean annual temperature of each grid cell to yield the temperature anomalies. A sensitivity analysis using a 1960–1990 baseline yielded very similar results.

Following approaches used in previous studies (20, 42), we calculated the anthropogenic climate change (ACC) signal using a 50-y moving average of the temperature anomaly in each grid cell of each model, combining the historical and RCP simulation, to remove high frequency variability. Because the signal across CMIP5 and CMIP6 models were statistically indistinguishable at our station sites, we used simulations from both sets of models in the analysis. We consider the observed temperature time-series from CRU to be the full forcing (with ACC) and subtracted the ACC signal from each of the 22 climate models from the observed temperature time-series to yield the no-ACC forcing scenario (i.e., a counter factual of temperature time-series at a site in a world without ACC-forced climate change; e.g., ref. 42).

To calculate the contribution of ACC to the observed pollen trends, we used the “best” mixed effects model selected above to predict each of the four pollen metrics as a function of temperature for each station and year. We then quantified the ACC contribution, estimated by climate model *i*, as the 1990–2018 or 2003–2018 trend predicted from with-ACC forced temperature (i.e., observed temperature; *m*_wACC,__i_) minus the trend predicted from no-ACC forced temperature (*m*_noACC,__i_), divided by the total observed trend (*m*_obs_):

$$\mathrm{Percent}\;\mathrm{ACC}\;\mathrm{contributio}n_{i}=100\times \frac{m_{\mathrm{wACC,}i}-m_{\mathrm{noACC,i}}}{m_{\mathrm{obs}}}.$$

We note that this approach and calculation is likely quite conservative and underestimates the full effects of ACC on pollen for several reasons. The potential strength of the ACC contribution is largely constrained by the predictive ability of the pollen-temperature model. Sources of uncertainty and imperfect predictive ability in that model, including temporal aggregation of climate and pollen data and the taxonomic aggregation of pollen data, will tend to “flatten” (i.e., decrease sensitivity and slope) the pollen-temperature predictions and decrease the ACC contribution. More complicated pollen-climate models, such as daily or weekly climate data, alternate climate variables like growing degree days, or modeling major pollen taxa as a function of different climate drivers, could increase the predictive ability of the pollen-climate models and, thus, the ACC contribution.

We also note that uncertainties and caveats remain, including potential nonclimate confounding factors, but our methods should largely minimize the impact of these factors. Because the spatial footprint of pollen stations is not well-understood and is likely temporally variable depending on weather conditions, nonclimate drivers could influence pollen trends but cannot be estimated currently. Long-term change in urban or peri-urban vegetation biomass or species composition due to land-use change or tree growth might influence pollen patterns. We tested for this using MODIS satellite data (MOD13A3-006) of the annual average EVI and NIRv 1-km resolution vegetation metrics over the 2000–2018 window and found no significant trends at our NAB stations, nor were vegetation metric trends significantly correlated with pollen trends across sites (*P* = 0.15; *SI Appendix*, Fig. S9). In addition, our ACC contribution analysis is based on interannual variation at individual sites and how that variation is related to climate, which would not be greatly affected by nonclimate trends. Furthermore, a positive secular nonclimate trend in pollen that is unrelated to temperature would act to decrease our estimated percent ACC contribution by increasing the denominator in the equation above. Thus, while these are certainly caveats, our analysis should be largely robust to these potential confounding factors and is likely a conservative estimate.

### Statistics.

We ensured that assumptions of normality and homogeneity of variances were met with Q-Q plots via the qqPlot diagnostic in the “car” R package (43, 44). Mixed effects models were conducted using the lme function in the “nlme” R package. Model selection was undertaken using the dredge function in the “MUMIn” R package. Climate observational data and model analysis were conducted using the “RNetCDF” R package, and maps were made using the “rworldmap” package. All analyses were conducted in the R statistical software (45).

## Supplementary Material

Supplementary File

## Data Availability

Data access can be requested from the National Allergy Bureau: https://www.aaaai.org/Aaaai/media/MediaLibrary/PDF%20Documents/NAB/NAB-Data-Release-Guidelines-Final-7-24-13.pdf.
