# Supplementary material for: Anthropogenic climate change is worsening North American pollen seasons
Source: Proc Natl Acad Sci U S A. 2021 Feb 8;118(7):e2013284118. doi: 10.1073/pnas.2013284118 (PMC7896283; doi:10.1073/pnas.2013284118)
Supplement: Supplementary File [file pnas.2013284118.sapp.pdf]

Anthropogenic climate change is worsening North American pollen seasons

Supplementary Tables

Table S1: List of the cities included in the study, number of years of data per city, and average number of observations per year at each station.

Station	Nyears	Ave.Nobs
1-Eugene	19	171.6
3-Seattle	16	182.6
4- Colorado Springs	16	242.3
9- Pleasanton	16	85.3
10- Roseville	19	46.9
11- Draper	16	128.4
14- Santa Barbara	7	135.4
24- Fargo	9	128.4
28- Onalaska (LaCrosse)	15	91.4
32- Minneapolis	9	168.3
33- Bellevue (Omaha)	29	267.5
35- St. Louis	19	247.3
41- Lexington	15	127.2
47- Tulsa	16	155.5
49- Marietta (Atlanta)	19	252.5
51- Baltimore	15	213.1
52- Charlotte	16	74.3
55- Huntsville	10	102.4
58- Tallahassee	5	229.0
59- Tampa	9	81.9
60- Silver Spring	19	198.4
62- Brooklyn	9	133.0
65- Erie	15	123.4
66- Olean	15	86.5
69- Rochester	15	159.2
85- Mountain View	16	48.7

86- Sparks (Reno)	16	51.6
94- Waterbury	15	124.8
97- Kansas City	15	168.5
100- College Station	16	232.3
102- Louisville	6	87.0
103- Oklahoma City	19	236.7
105- Waco (Station 1)	16	243.3
106- Waco (Station 2)	19	234.7
110- London	18	235.3
111- Austin Area (Georgetown)	16	251.2
134- Albany	9	196.9
138- Savannah	16	45.3
141- Niagara Falls	8	184.4
143- Baton Rouge	5	74.5
146- Armonk	15	137.3
151- Madison	15	118.5
154- Greenville	16	197.6
157- York	15	150.6
159- San Diego	16	342.4
167- Houston (Station 1)	5	154.6
172- Stockton	7	137.5
181- Flower Mound	12	352.7
188- Houston (Station 2)	11	213.7
189- New York	9	171.0
192- Birmingham	10	230.9
194- Springfield	9	160.6
196- Denver	8	144.9
198- San Antonio	9	322.8
202- Asheville	9	143.4
203- Midland	8	53.9
213- Northern Kentucky (Cincinnati)	6	75.0
Fairbanks, AK	19	-
Winnipeg, Canada	23	-
Saskatoon, Canada	22	-

Table S2: Temporal trends in total annual integrals using different thresholds of the minimum number of stations. Displayed is the slope of log-transformed annual pollen integrals against time and the p-value of the mixed effects model.

Model	Slope	p-value
All station-years	0.007	<0.0001
Years with >5 stations	0.005	<0.0001
Years with >40 stations	0.004	0.005

Table S3: Temporal trends in 10 pollen season metrics. Displayed is the slope of log-transformed pollen metrics against time, back-transformed change over 1990-2018 in % for concentrations and days for start date, end date, and season length metrics, and the p-value of the mixed effects model. *=variable where square-root transform was needed instead of log-transformation.

Pollen metric	Slope	Change	p-value
Maximum daily count	0.009	+24%	<0.0001
Mean daily count	0.005	+14.8%	<0.0001
Median daily count	0.002	+5.7%	0.1
Season start date*	-0.015	-20d	0.01
Season end date	-0.0001	-1d	0.1
Season length	0.0008	+8d	0.0003
Spring total count	0.008	+21.5%	<0.0001
Summer total count	-0.004	-11.4%	0.006
Fall total count	-0.001	-3.3%	0.44
Annual total count	0.007	+20.9%	<0.0001

Table S4: Model selection results for climate-pollen models. Model formula, Akaike Information Criterion (AIC), and marginal and conditional R^2 value for the mixed effects model.

Model	AIC	$R^2_{marginal}$	$R^2_{conditional}$
AnnualCount ~ CO2	-2655.6	0.01	0.9
AnnualCount ~ CO2 + AnnualTemp	-2654.7	0.149	0.88
AnnualCount ~ AnnualTemp	-2652.9	0.2	0.87
SpringCount ~ CO2 + AnnualTemp	-1969.6	0.11	0.85
SpringCount ~ AnnualTemp	-1968.6	0.14	0.83
StartDate ~ SpringTemp	2522	0.25	0.67
StartDate ~ AnnualTemp	2522.6	0.37	0.71
SeasonLength ~ AnnualTemp	2418	0.21	0.51

Table S5: Earth system models included in this analysis.

Model	Center	CMIP5/6	Reference
ACCESS1-0	Commonwealth Scientific and Industrial Research Organisation, Bureau of Meteorology	CMIP5	(*1*)
ACCESS1-3	Commonwealth Scientific and Industrial Research Organisation, Bureau of Meteorology	CMIP5	(*1*)
CanESM2	Canadian Centre for Climate Modelling and Analysis	CMIP5	(*2*)
Mk3-6-0	Commonwealth Scientific and Industrial Research Organisation	CMIP5	(*3*)
INM CM4	Institute for Numerical Mathematics	CMIP5	(*4*)
CM5A-LR	Institut Pierre Simon Laplace	CMIP5	(*5*)
CM5A-MR	Institut Pierre Simon Laplace	CMIP5	(*5*)
CM5B-LR	Institut Pierre Simon Laplace	CMIP5	(*5*)
MIROC-ESM	Japan Agency for Marine-Earth Science and Technology (JAMSTEC) and Centre for Climate System Research / National Institute for Environmental Studies, Japan	CMIP5	(*6*)
MIROC5	Japan Agency for Marine-Earth Science and Technology (JAMSTEC) and Centre for Climate System Research / National Institute for Environmental Studies, Japan	CMIP5	(*6*)
MPI-ESM-LR	Max Plank Institute for Meteorology	CMIP5	(*7*)
MPI-ESM-MR	Max Plank Institute for Meteorology	CMIP5	(*7*)
MRI-CGCM3	Meteorological Research Institute (MRI) of Japan	CMIP5	(*8*)
MRI-ESM1	Meteorological Research Institute (MRI) of Japan	CMIP5	(*8*)
CSM2-MR	Beijing Climate Center	CMIP6	(*9*)
ESM1	Beijing Climate Center	CMIP6	(*10*)
CAMS-CSM1	Chinese Academy of Meteorological Sciences	CMIP6	(*11*)
CanESM5	Canadian Centre for Climate Modelling and Analysis	CMIP6	(*12*)
CESM2	National Center for Atmospheric Research	CMIP6	(*13*)
CM6A-LR	Institut Pierre Simon Laplace	CMIP6	(*14*)
MIROC6	Japan Agency for Marine-Earth Science and Technology (JAMSTEC) and Centre for Climate System Research / National Institute for Environmental Studies, Japan	CMIP6	(*15*)
MRI-ESM2	Meteorological Research Institute (MRI) of Japan	CMIP6	(*16*)

Supplementary Figures

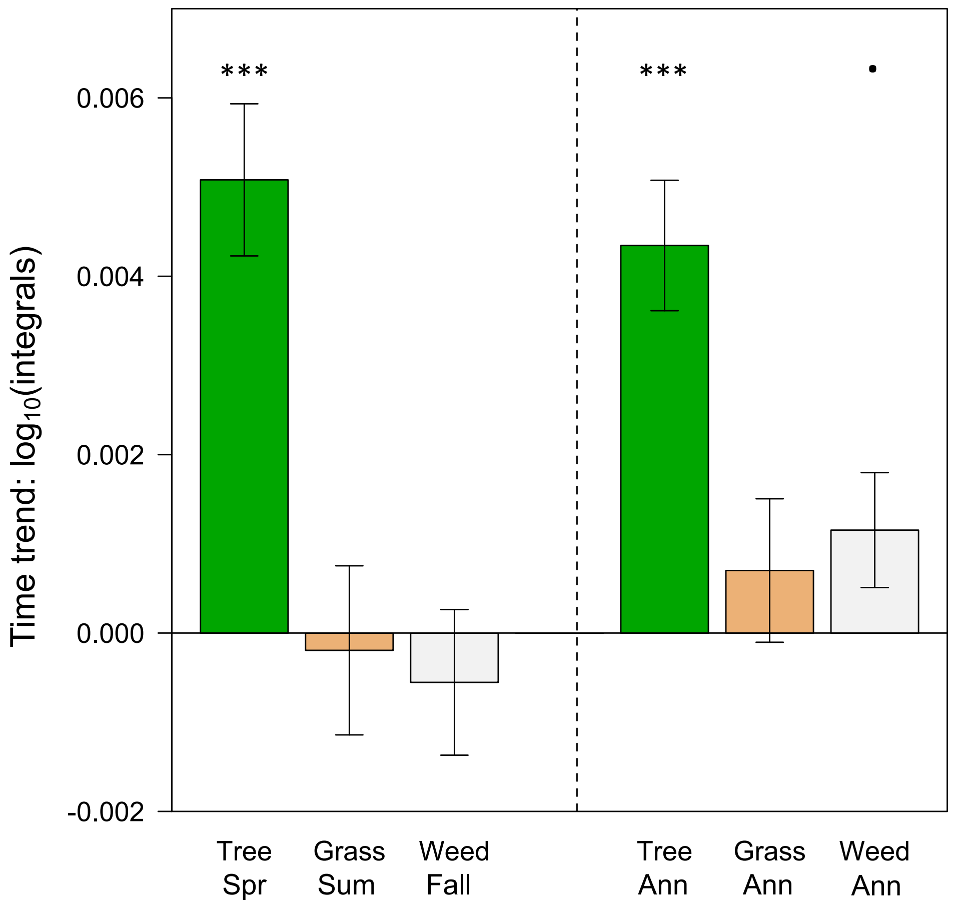

Figure S1: Temporal trend in pollen integrals for tree, grass, and weed taxa for specific seasons (left) and annual integrals (right). Error bars indicate +/- 1 standard error. '***' indicates $p<0.001$ and '.' indicates p-values between 0.05-0.1.

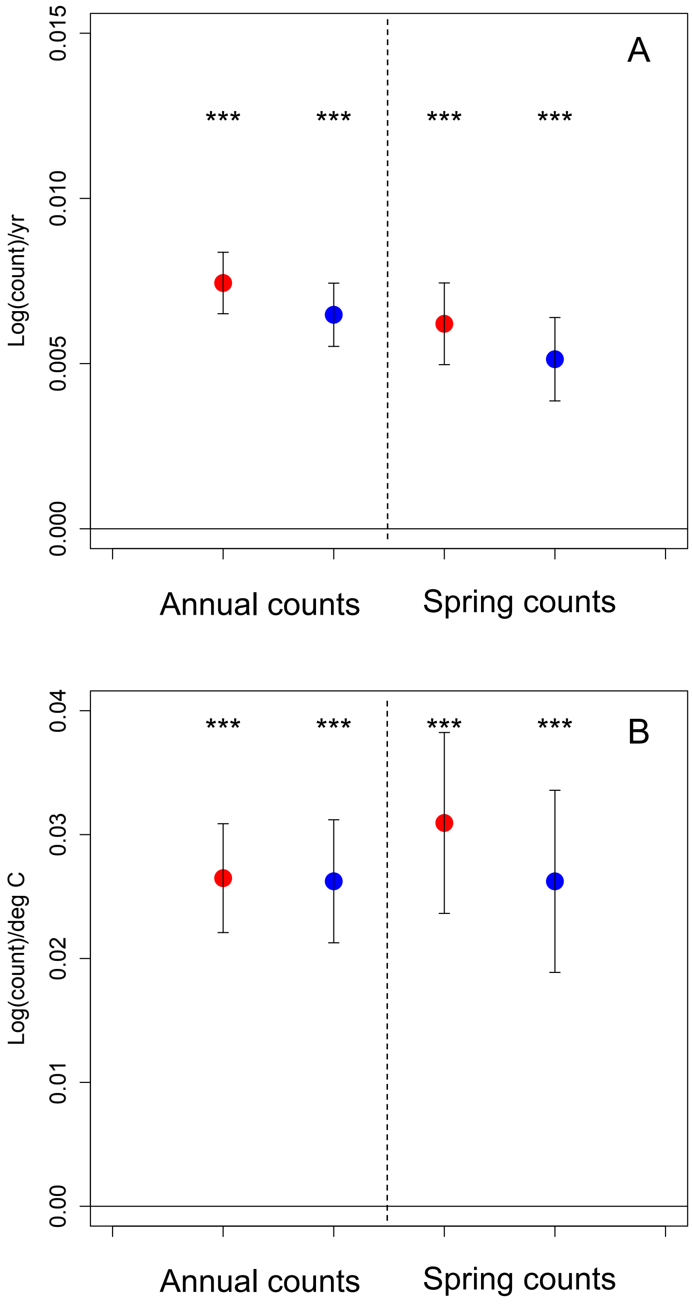

Figure S2: Temporal trends (A) and temperature sensitivities (B) in pollen stations are robust when examining all stations (red) versus only stations with 10+ years of data (blue).

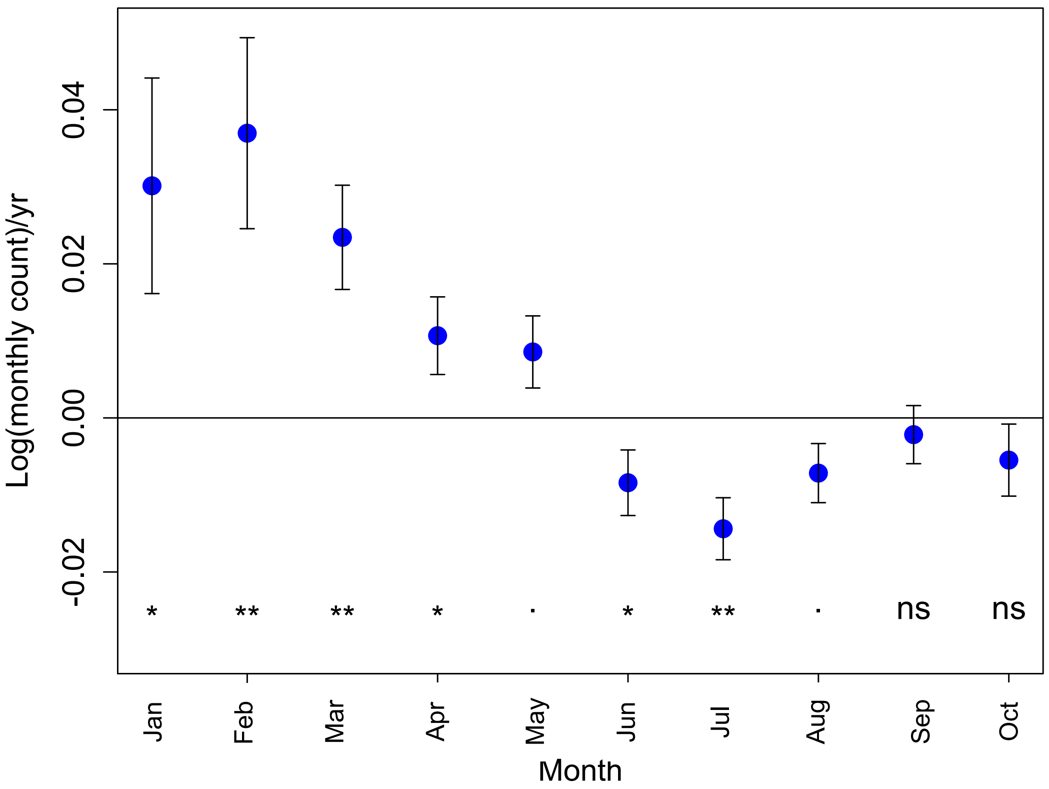

Figure S3: Trends in monthly integral data across all stations over the 1990-2018 period. November and December did not have enough station-year data to estimate robust trends. Statistics: *$p<0.05$, **$p<0.01$

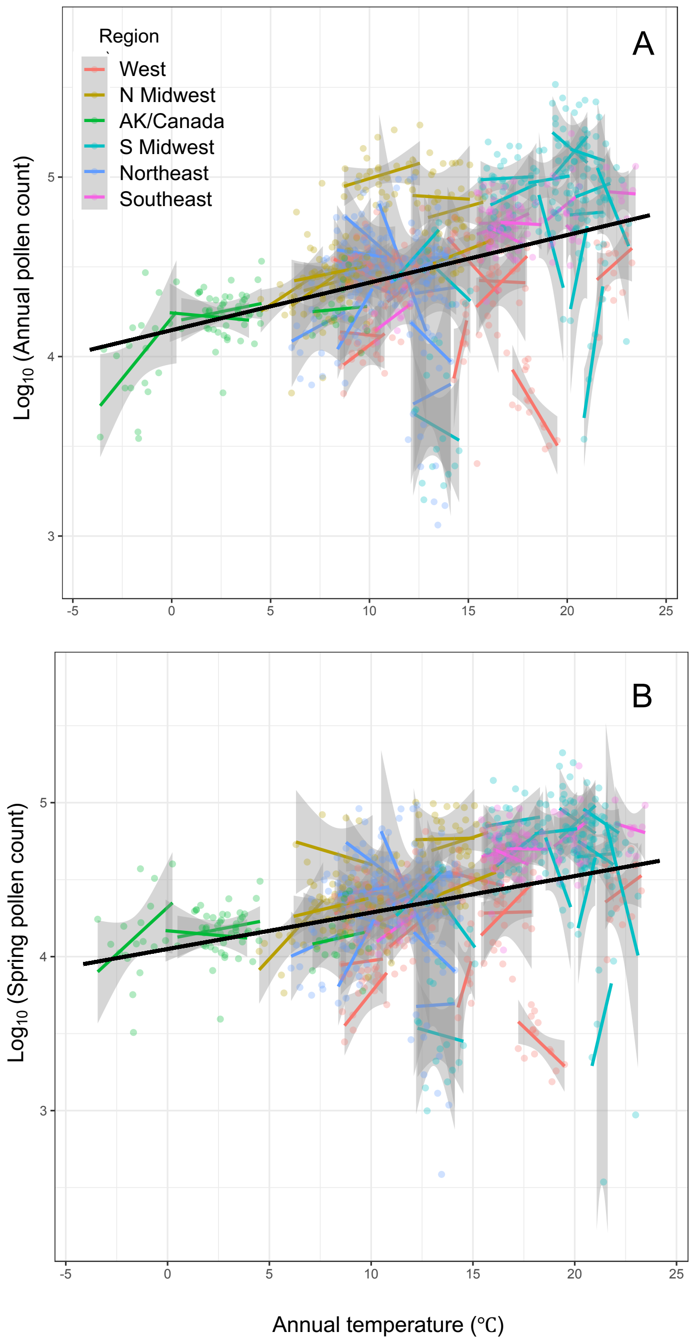

Figure S4: Pollen-climate relationships with individual years and cities for annual pollen integrals (A) and spring pollen integrals (B). Colors indicate region of the country (as in Fig. S6). Black line is the fixed effect of annual temperature estimated across all sites from the mixed effects model.

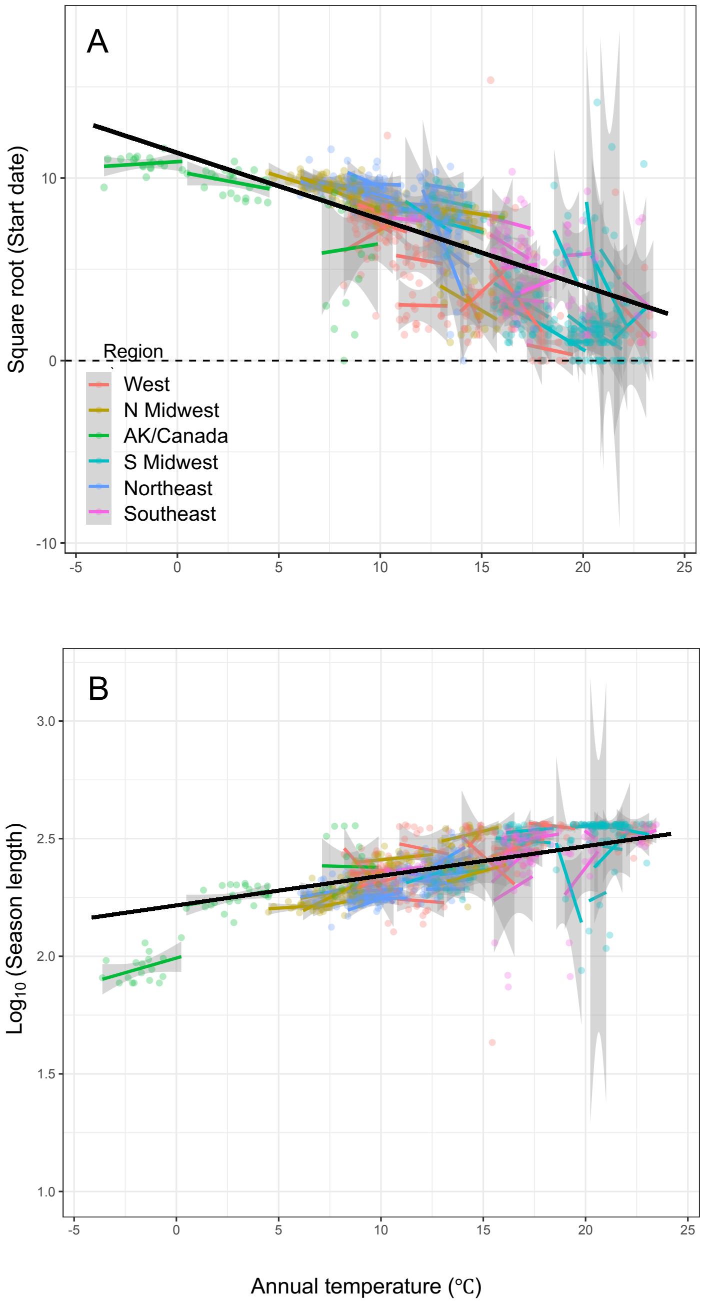

Figure S5: Pollen-climate relationships with individual years and cities for pollen season start date (A) and pollen season length (B). Colors indicate region of the country (as in Fig. S6). Black line is the fixed effect of annual temperature estimated across all sites from the mixed effects model.

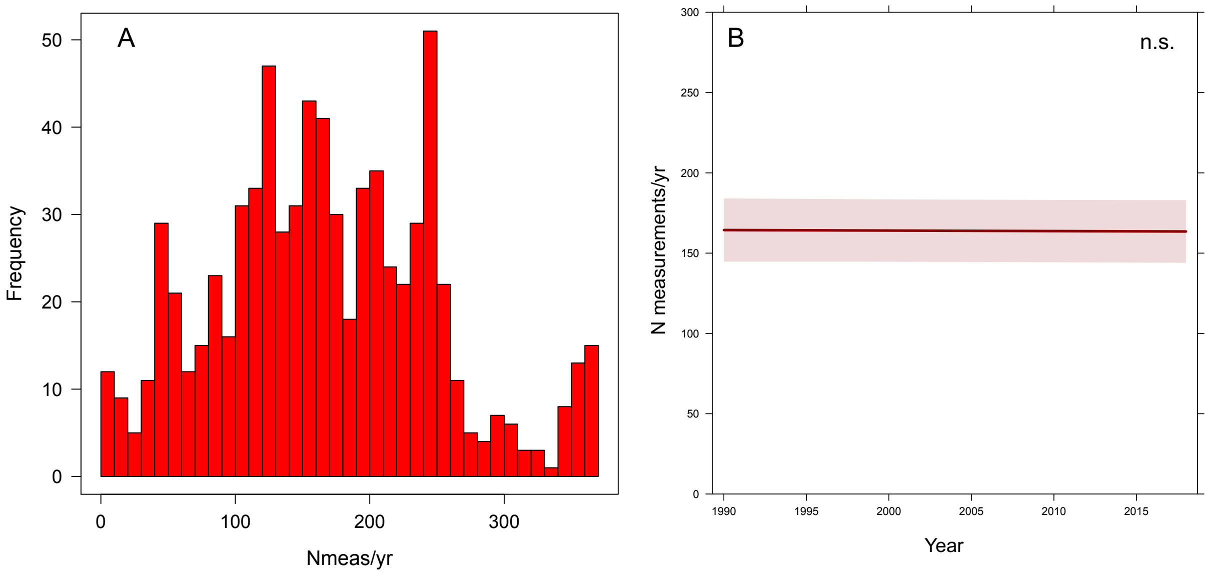

Figure S6: (A) Histogram of the number of pollen observations per year (Nmeas/yr) across all stations with daily pollen data (Table S1) in the study. (B) Temporal trend of the number of pollen observations per year across all stations with daily pollen data (Table S1) in the study.

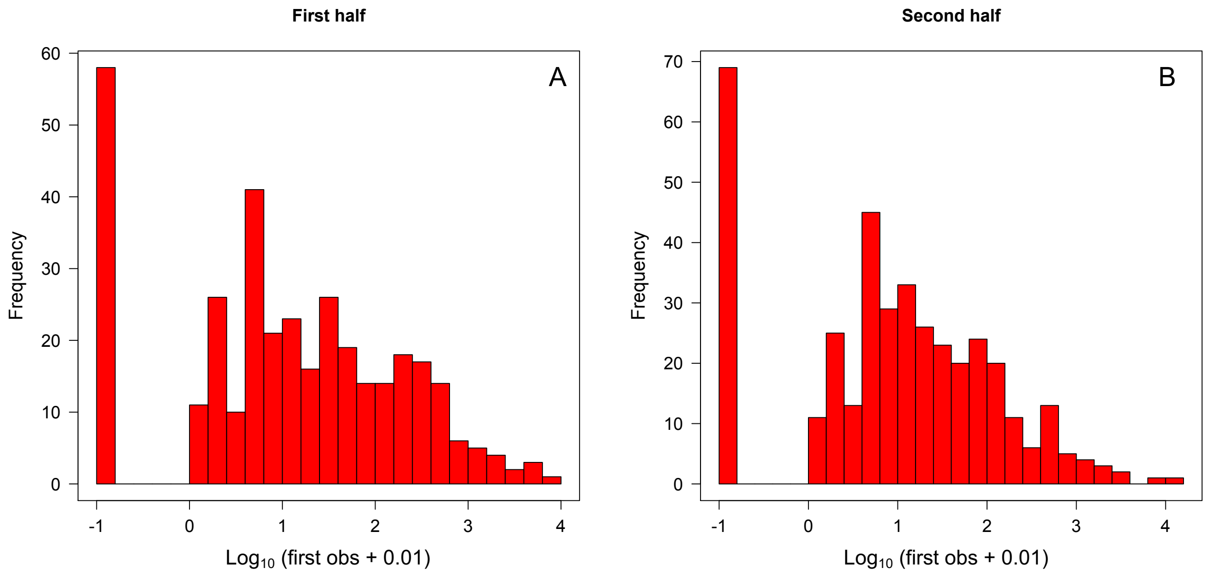

Figure S7: Initial pollen concentrations are largely similar between the first (A) and second (B) halves of station's records. Log_{10} +0.01 of the initial measurement in a year are shown and thus a measurement of zero is plotted as a value of -1.

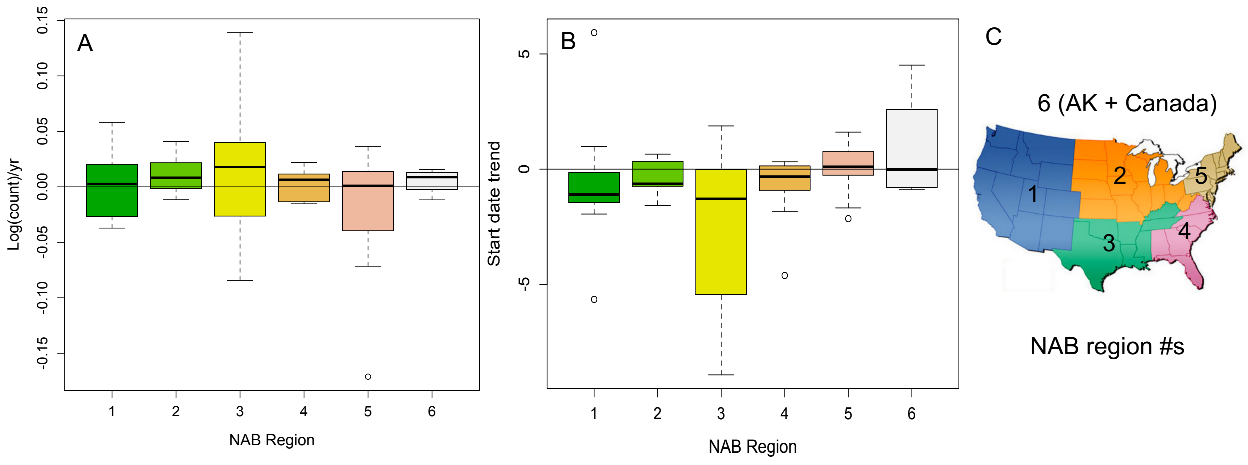

Figure S8: Regional patterns in pollen metric trends. (A) Boxplot of trends in annual pollen counts. (B) Boxplot of trends in pollen season start date. (C) National Allergy Bureau (NAB) region numbers plus a sixth region added here for Alaska and Canada stations.

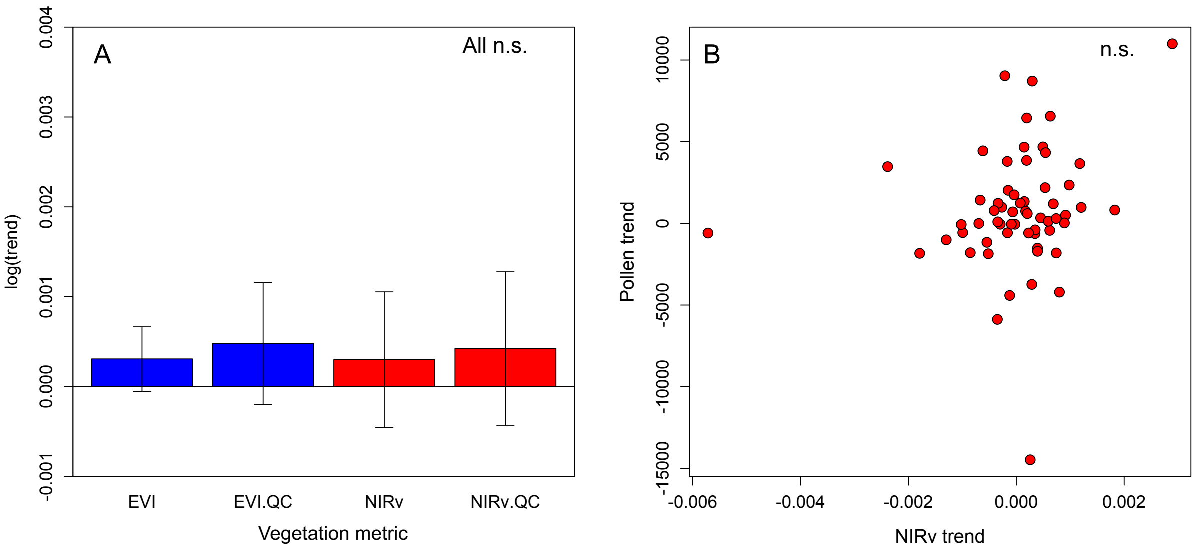

Figure S9: (A) Temporal trend from 2000-2018 of the grid cells covering all pollen stations in this analysis using the enhanced vegetation index (EVI) and near-infrared reflectance of vegetation (NIRv) from MODIS data, using all data and a quality-controlled (QC) subset of data. Error bars indicate the 95% confidence interval. All trends were non-significant ($p>0.11$). (B) Temporal trends in annual pollen concentrations and annual average NIRv values for all pollen stations in the analysis (linear regression p-value = 0.15).

References

1. M. Collier, P. Uhe, *CMIP5 datasets from the ACCESS1. 0 and ACCESS1. 3 coupled climate models* (Citeseer, 2012).

2. P. Chylek, J. Li, M. K. Dubey, M. Wang, G. Lesins, Observed and model simulated 20th century Arctic temperature variability: Canadian earth system model CanESM2. *Atmospheric Chem. Phys. Discuss.* **11**, 22893–22907 (2011).

3. M. A. Collier, S. J. Jeffrey, L. D. Rotstayn, K. K. Wong, S. M. Dravitzki, C. Moseneder, C. Hamalainen, J. I. Syktus, R. Suppiah, J. Antony, in *International Congress on Modelling and Simulation–MODSIM* (2011).

4. E. M. Volodin, N. A. Dianskii, A. V. Gusev, Simulating present-day climate with the INMCM4. 0 coupled model of the atmospheric and oceanic general circulations. *Izv. Atmospheric Ocean. Phys.* **46**, 414–431 (2010).

5. J. Mignot, S. Bony, Presentation and analysis of the IPSL and CNRM climate models used in CMIP5. *Clim Dyn*. **40**, 2089 (2013).

6. S. Watanabe, T. Hajima, K. Sudo, T. Nagashima, T. Takemura, H. Okajima, T. Nozawa, H. Kawase, M. Abe, T. Yokohata, MIROC-ESM 2010: Model description and basic results of CMIP5-20c3m experiments. *Geosci. Model Dev.* **4**, 845 (2011).

7. M. Giorgetta, et al., CMIP5 simulations of the Max Planck Institute for Meteorology (MPI-M) based on the MPI-ESM-LR model: The rcp85 experiment, served by ESGF (2012), doi:doi:10.1594/WDCC/CMIP5.MXELr8.

8. Y. Adachi, et al., MRI-ESM1 model output prepared for CMIP5 historical, served by ESGF (2015), doi:doi:10.1594/WDCC/CMIP5.MRM1hi.

9. X. Xin, J. Zhang, F. Zhang, T. Wu, X. Shi, J. Li, M. Chu, Q. Liu, J. Yan, Q. Ma, M. Wei, BCC BCC-CSM2MR model output prepared for CMIP6 CMIP (2018), , doi:10.22033/ESGF/CMIP6.1725.

10. J. Zhang, T. Wu, X. Shi, F. Zhang, J. Li, M. Chu, Q. Liu, J. Yan, Q. Ma, M. Wei, BCC BCC-ESM1 model output prepared for CMIP6 CMIP historical (2018), , doi:10.22033/ESGF/CMIP6.2949.

11. X. Rong, CAMS CAMS_CSM1.0 model output prepared for CMIP6 CMIP historical (2019), , doi:10.22033/ESGF/CMIP6.9754.

12. N. C. Swart, J. N. Cole, V. V. Kharin, M. Lazare, J. F. Scinocca, N. P. Gillett, J. Anstey, V. Arora, J. R. Christian, S. Hanna, The Canadian Earth System Model version 5 (CanESM5. 0.3). *Geosci. Model Dev.* **12**, 4823–4873 (2019).

13. G. Danabasoglu, NCAR CESM2 model output prepared for CMIP6 CMIP historical (2019), , doi:10.22033/ESGF/CMIP6.7627.

14. O. Boucher, S. Denvil, A. Caubel, M. A. Foujols, IPSL IPSL-CM6A-LR model output prepared for CMIP6 CMIP (2018), , doi:10.22033/ESGF/CMIP6.1534.

15. H. Tatebe, M. Watanabe, MIROC MIROC6 model output prepared for CMIP6 CMIP (2018), , doi:10.22033/ESGF/CMIP6.881.

16. S. Yukimoto, T. Koshiro, H. Kawai, N. Oshima, K. Yoshida, S. Urakawa, H. Tsujino, M. Deushi, T. Tanaka, M. Hosaka, H. Yoshimura, E. Shindo, R. Mizuta, M. Ishii, A. Obata, Y. Adachi, MRI MRI-ESM2.0 model output prepared for CMIP6 CMIP (2019), , doi:10.22033/ESGF/CMIP6.621.
